# Exploring the Hyperspectral Response of Quercetin in *Anoectochilus roxburghii* (Wall.) Lindl. Using Standard Fingerprints and Band-Specific Feature Analysis

**DOI:** 10.3390/plants14203141

**Published:** 2025-10-11

**Authors:** Ziyuan Liu, Haoyuan Ding, Sijia Zhao, Hongzhen Wang, Yiqing Xu

**Affiliations:** 1 College of Optical, Mechanical and Electrical Engineering, Zhejiang A&F University, Hangzhou 311300, China; liuziyuan@zafu.edu.cn (Z.L.); dhytiaspetto@outlook.com (H.D.); 2 College of Food and Health, Zhejiang A&F University, Hangzhou 311300, China; zhaosijia0322@163.com

**Keywords:** hyperspectral imaging, *Anoectochilus roxburghii*, quercetin, spectral fingerprinting, SPA, 1D-CNN, metabolite detection, medicinal plant

## Abstract

Quercetin, a key flavonoid in *Anoectochilus roxburghii* (Wall.) Lindl., plays an important role in determining the pharmacological value of this medicinal herb. However, traditional methods for quercetin quantification are destructive and time-consuming, limiting their application in real-time quality monitoring. This study investigates the hyperspectral response characteristics of quercetin using near-infrared hyperspectral imaging and establishes a feature-based model to explore its detectability in *A. roxburghii* leaves. We scanned standard quercetin solutions of known concentration under the same imaging conditions as the leaves to produce a dilution series. Feature-selection methods used included the successive projections algorithm (SPA), Pearson correlation, and competitive adaptive reweighted sampling (CARS). A 1D convolutional neural network (1D-CNN) trained on SPA-selected wavelengths yielded the best prediction performance. These key wavelengths—particularly the 923 nm band—showed strong theoretical and statistical relevance to quercetin’s molecular absorption. When applied to plant leaf spectra, the standard-trained model produced continuous predicted quercetin values that effectively distinguished cultivars with varying flavonoid contents. PCA visualization and ROC-based classification confirmed spectral transferability and potential for functional evaluation. This study demonstrates a non-destructive, spatially resolved, and biochemically interpretable strategy for identifying bioactive markers in plant tissues, offering a methodological basis for future hyperspectral inversion studies and intelligent quality assessment in herbal medicine.

## 1. Introduction

*Anoectochilus roxburghii* (Wall.) Lindl., commonly known as “Jinxianlian” in Chinese, is a perennial medicinal herb belonging to the Orchidaceae family [1]. It has long been used in Traditional Chinese Medicine for its diverse pharmacological effects, including hepatoprotective, anti-inflammatory, antihyperglycemic, and immunoregulatory activities [2,3]. Its medicinal value has attracted increasing attention in recent years, both for clinical application and for the development of high-value herbal products [4]. These therapeutic properties are attributed primarily to its rich content of bioactive secondary metabolites, including polysaccharides, flavonoids, glycosides, and phenolic acids [5]. Among these, flavonoids—especially quercetin and its glycosides—have been widely recognized as major active constituents and quality markers of *A. roxburghii* [6,7]. Quercetin is a widely distributed flavonol in plants and is known for its strong antioxidant, anti-inflammatory, antiviral, and anti-tumor effects [8,9]. Due to its well-established health benefits and chemical stability, quercetin is often selected as a representative compound for evaluating flavonoid content in herbal materials [10]. In *A. roxburghii*, studies have shown that quercetin plays a key role in regulating the pharmacological efficacy and is closely related to the functional differences observed among different cultivars [11]. Therefore, monitoring the distribution and variation of quercetin in *A. roxburghii* has become a crucial step in germplasm screening, cultivation management, and quality control. Currently, the quantification of quercetin in plant materials is primarily based on chromatographic methods, especially high-performance liquid chromatography (HPLC) equipped with ultraviolet (UV) or mass spectrometry (MS) detectors [12,13,14]. Although these methods offer high accuracy and sensitivity, they suffer from several inherent limitations. First, HPLC requires destructive sampling, complex pretreatment procedures (extraction, centrifugation, filtration), and the use of organic solvents. Second, these assays are time-consuming and labor-intensive, limiting their applicability in large-scale, rapid, or in situ screening tasks [15]. In breeding and cultivation applications where rapid decision-making is required, traditional HPLC methods are impractical. Furthermore, HPLC cannot provide spatial distribution information of the compound within plant tissues, which is increasingly demanded in precision agriculture and functional metabolomics.

To address these limitations, optical sensing technologies such as near-infrared (NIR) spectroscopy and hyperspectral imaging (HSI) have emerged as promising non-destructive alternatives [16,17]. NIR spectroscopy leverages the absorption characteristics of overtones and combination bands from functional groups (mainly O–H, C–H, and N–H) in the 800–2500 nm range, enabling rapid and reagent-free compound identification [18]. However, conventional NIR systems typically acquire only single-point spectra, lacking spatial resolution. In contrast, hyperspectral imaging combines traditional spectroscopy with imaging, capturing both spatial and spectral information in a three-dimensional datacube (x, y, λ). This enables simultaneous assessment of chemical composition and spatial distribution across the sample surface [19,20]. Hyperspectral imaging has demonstrated significant potential in the field of medicinal plant analysis. Recent studies have applied HSI to detect total flavonoids, polysaccharides, and alkaloids in herbs such as Chrysanthemum morifolium, Lycium barbarum, and Panax notoginseng, achieving satisfactory results for quality classification and content estimation [21,22,23]. Recent studies demonstrate that NIR-HSI, when combined with appropriate preprocessing, wavelength selection and either multivariate or deep-learning regressors, can non-destructively quantify quercetin and reveal its spatial distribution in plant materials. For example, Kusumiyati et al. reported strong PLSR performance for carotenoids and quercetin in Tagetes erecta using a handheld Vis–NIR probe [24]. He et al. applied NIR-HSI with wavelength-selection and modeling to Chrysanthemum morifolium, reporting good predictive metrics for flavonoids [25].

Despite these advances, most studies apply hyperspectral techniques mainly to predict bulk chemical composition using empirical regression models, rather than to probe the intrinsic spectral response of individual bioactive compounds. For quercetin, previous work [23,24,25] demonstrated the feasibility of quantification using near-infrared hyperspectral imaging coupled with deep-learning regression; however, that study emphasized concentration prediction and quality control and did not interrogate the chemical mechanisms behind informative bands or the spectral consistency across samples. As a result, mechanistic understanding of how key compounds such as quercetin modulate the reflectance of fresh plant tissues remains limited, which reduces model interpretability and constrains the deployment of hyperspectral methods for compound-specific, non-destructive detection. Moreover, few studies have addressed the spectral consistency or transferability between pure standard solutions and their corresponding metabolites in intact plant tissues. Given the structural complexity and heterogeneity of biological matrices, it is unclear whether the spectral patterns observed in isolated compounds remain detectable when those compounds are integrated into leaf tissues. Understanding this relationship is critical for advancing spectral fingerprinting methodologies, enhancing model robustness, and supporting the development of targeted, interpretable models for in vivo compound detection.

In this study, we systematically interrogate the near-infrared hyperspectral response of quercetin and directly link those responses to intact leaves of *A*. *roxburghii*. Rather than treating hyperspectra only as inputs to black-box concentration predictors, we use a feature-driven workflow that scans a quercetin dilution series and leaves under identical imaging conditions and then combines complementary diagnostics—sparse wavelength selection (SPA, CARS), Pearson correlation, PLSR with VIP scoring, unsupervised spectral decomposition (NMF), derivative preprocessing, and 1D-CNN feature learning—to attribute specific NIR bands to quercetin. These cross-method checks consistently identify a small set of concentration-related bands (the strongest single band at ~923.0 nm, VIP = 2.93) and yield a robust standard–leaf Pearson correlation (r ≈ 0.75). By explicitly bridging pure-compound spectra and intact-tissue reflectance, the work provides mechanistic evidence for band assignment, improves model interpretability, and lays a practical foundation for non-destructive, spatially resolved quercetin mapping and subsequent quantitative inversion in medicinal plants. This approach moves hyperspectral applications beyond bulk indices toward compound-specific, interpretable detection that is readily applicable to high-throughput phenotyping and digital quality assessment.

## 2. Results and Discussion

### 2.1. Spectral Response and Feature Selection Analysis

**Figure 1:**
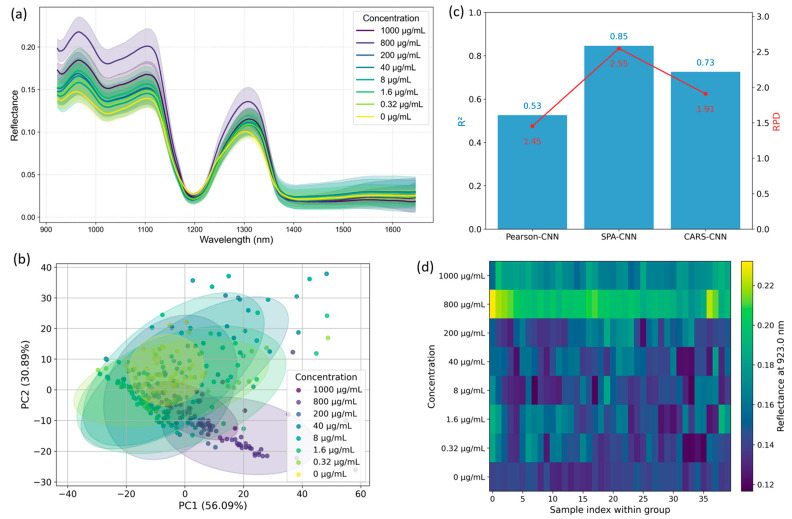
Exploratory analysis of hyperspectral responses for quercetin standard solutions. (**a**) Mean reflectance spectra with shaded area representing ± SD. (**b**) PCA score plot illustrating overall sample distribution. (**c**) Comparison of three feature selection methods combined with 1D-CNN models: Pearson correlation, SPA, and CARS. (**d**) False-color image based on a representative SPA-selected wavelength, visualizing spatial variations linked to concentration.

To investigate whether HSI can sensitively capture the presence and variation of functional metabolites in medicinal plants, we conducted a systematic analysis of the spectral response of quercetin standards across a wide concentration range (0–1000 μg/mL). Rather than pursuing precise quantification, this study explores the spectral detectability and characteristic responses of quercetin in the near-infrared region. We aim to determine whether specific wavelengths or spectral patterns can serve as optical fingerprints of this flavonoid. Figure 1 summarizes the key findings of this exploratory analysis. As shown in Figure 1a, the mean reflectance spectra (±SD) of eight quercetin concentration levels exhibit broad similarity, with only subtle intensity variations in the 900–1700 nm range. Importantly, no clearly distinguishable absorption peaks or concentration-correlated spectral trends are visible, especially at lower concentrations. This suggests that human-observable differences in raw spectra are limited and may obscure important chemical cues. To investigate whether the high-dimensional spectral data harbor latent structures related to concentration, PCA was performed (Figure 1b). The resulting score plot shows only modest separation among concentration groups along the first two principal components, and substantial overlap is observed for intermediate and low concentrations. This further illustrates the difficulty of interpreting spectral signals through unsupervised or low-dimensional visual methods.

Given the limitations of direct observation and global projections, we applied three wavelength selection strategies—Pearson correlation, SPA, and CARS—to extract bands most sensitive to concentration changes. These subsets were used as input to a 1D-CNN model, and predictive performance was assessed via five-fold cross-validation. As shown in Figure 1c, under our five-fold cross-validation scheme, the SPA-based 1D-CNN produced the best internal performance (R^2^ = 0.833, RPD = 2.45) compared with the tested alternatives; these metrics are indicative of relative model behaviour on our standard series but should not be interpreted as absolute, instrumentation-independent performance benchmarks. To assess the interpretability of these bands, we examined the correlation between each SPA-selected wavelength and quercetin concentration. The results, summarized in Table 1, reveal that several SPA-identified bands exhibit strong positive correlations with concentration, particularly the 923.0 nm band (r = 0.696), which also appeared as the most sensitive feature in this set. Notably, this wavelength aligns precisely with the one used in the false-color spatial map in Figure 1d, reinforcing its biological relevance and modeling significance. We further visualized sample reflectance distributions at this band (923.0 nm) using a false-color intensity map (Figure 1d). Although raw spectra (Figure 1a) lacked clearly separable features, this band enabled an intuitive visual differentiation of concentration gradients. The resulting pattern revealed fine-scale spatial variation in quercetin-associated reflectance that would otherwise be imperceptible, highlighting the capability of HSI to identify subtle biochemical signatures.

**Figure 2:**
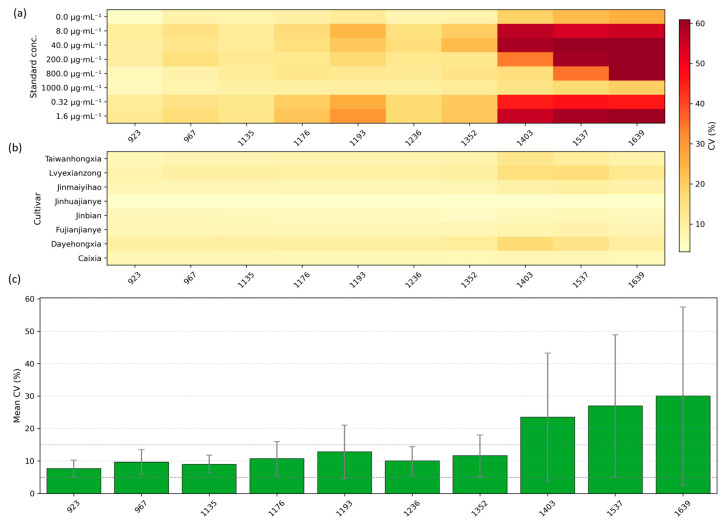
CV analysis of SPA-selected wavelengths. (**a**) CV distributions for eight quercetin standard concentration groups; (**b**) CV distributions for eight *A. roxburghii* cultivars; (**c**) mean CV across all groups for each SPA-selected wavelength (error bars = ±SD).

To provide a concise summary of measurement repeatability across wavelengths and sample types, we computed the CV for each SPA-selected wavelength separately for the standard quercetin solutions and for the eight cultivars, and summarized the results in Figure 2. Figure 2a displays the CV distribution for the eight standard concentration groups, while Figure 2b shows the CV distribution for the cultivars. Figure 2c presents the mean CV across all groups for each SPA-selected wavelength (error bars denote ±SD across groups). Overall, the majority of SPA-selected wavelengths show low variability (CV ≤ 5%), indicating good within-group repeatability under our acquisition protocol. A small number of wavelengths exhibit larger CVs, typically associated with very low mean reflectance or saturation effects at concentration extremes, and thus warrant cautious interpretation. 923 nm exhibited low CV and small inter-group variability under our acquisition protocol and was therefore chosen as a candidate representative band for downstream analyses; this choice is data-driven and specific to our instrument, preprocessing and sample set, and may not generalize across all sensors or tissues without further calibration. Collectively, these findings support the use of data-driven band selection as a bridge between machine learning prediction and mechanistic interpretation. By shifting focus from black-box modeling to spectral fingerprint analysis, this approach establishes a foundation for future studies on interpretable, sensor-based detection of plant metabolites. To explore the applicability of these findings in real plant tissues, we next investigate the hyperspectral responses of *A. roxburghii* leaves and evaluate the spectral similarities between standard quercetin solutions and in vivo plant samples.

### 2.2. Traditional Machine Learning Models for Goldthread Classification

**Figure 3:**
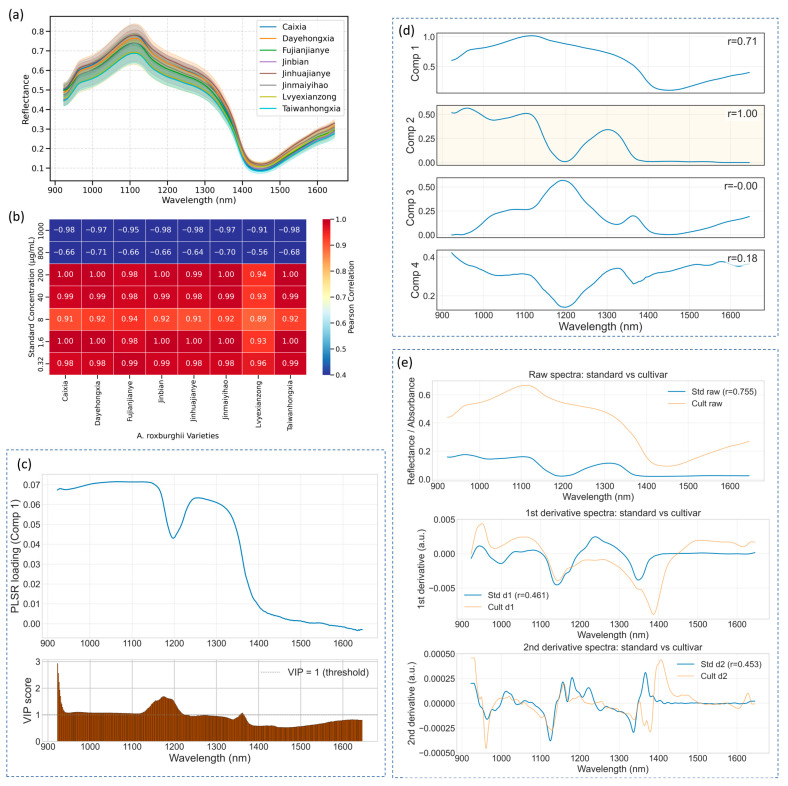
Spectral characteristics and cross-method similarity analysis of *A. roxburghii* leaves. (**a**) Mean reflectance spectra (±SD) of eight cultivars. (**b**) Correlation heatmap between leaf spectra and quercetin standards using SPA-selected wavelengths. (**c**) PLSR component-1 loadings (top) and VIP scores (bottom) from the standard dilution series (dashed line = VIP 1; strongest band 923.0 nm, VIP = 2.93). (**d**) NMF-recovered component spectra from leaf data; one component most closely matches the quercetin standard. (**e**) Comparison of raw, first-derivative and second-derivative preprocessing.

Figure 3a presents the mean reflectance spectra (±standard deviation) of eight *A. roxburghii* cultivars measured in the 900–1700 nm range. While minor spectral variations are observed—particularly in the shortwave infrared region—the overall spectral signatures remain highly similar across varieties, with no distinct absorption features that allow for intuitive visual differentiation. This underscores a fundamental limitation of using raw spectra for classification: the high dimensionality and subtle differences in reflectance often mask meaningful biochemical variation. In our previous study [26], we systematically addressed this challenge using machine learning techniques and demonstrated that accurate cultivar classification is feasible when combined with optimized feature selection and modeling strategies. Therefore, the present work does not revisit the classification task. Instead, we focus on elucidating spectral mechanisms, identifying quercetin-related absorption features, and evaluating their transferability from standard compounds to complex plant tissues.

To further evaluate the spectral resemblance between *A. roxburghii* leaves and quercetin standards, we conducted a similarity analysis using the SPA-selected wavelengths identified in Table 1. Rather than employing the full spectral range or threshold-filtered correlation bands, we utilized the same subset of informative bands that previously yielded the highest CNN prediction accuracy (Section 2.1). For each standard concentration, background-corrected net absorbance spectra were averaged and compared with the corresponding leaf spectra using Pearson correlation. The resulting correlation coefficients are summarized in Figure 3b. Interestingly, plant samples compared to standard solutions in the medium-to-low concentration range (200 μg/mL, 40 μg/mL, 8 μg/mL, 1.6 μg/mL, 0.32 μg/mL) exhibited consistently high positive correlations (r > 0.90) across most cultivars, with values in some cases exceeding 0.99. While these high correlations indicate shared spectral variance at the selected wavelengths in our dataset, they do not by themselves prove exclusive attribution to quercetin—co-occurring compounds, matrix effects and scattering may also contribute, so we interpret these results as preliminary evidence of transferability rather than definitive chemical assignment. In contrast, spectra from the highly concentrated standard groups (1000 μg/mL and 800 μg/mL) showed significantly diminished or even negative correlations, with the 1000 μg/mL group yielding values below –0.90 across all varieties. This inverse relationship may stem from nonlinear saturation effects at high quercetin concentrations, where excessive absorption causes broad reflectance suppression across the NIR region. Such exaggerated spectral depressions are not typically observed in plant tissues, leading to spectral divergence and negative correlation.

To understand the molecular basis of this correspondence, we further examined the spectral response at 923 nm—identified as the most informative band by SPA and also featured prominently. This wavelength lies within a region of the near-infrared spectrum dominated by overtone and combination bands arising from molecular vibrations of O–H, C–H, and N–H bonds [27,28]. The 923 nm band, in particular, corresponds to second overtone or combination vibrations of hydroxyl (O–H) groups, which are abundant in polyhydroxy flavonoids such as quercetin [29]. Its strong and specific absorbance response is influenced by both the number and microenvironment of O–H groups in the molecule [30]. Additionally, interactions between vibrational modes and plant tissue microstructure—such as water content and cellular arrangement—can amplify O–H-related absorption features, further enhancing sensitivity at 923 nm. These factors collectively render 923 nm a robust spectral fingerprint for detecting quercetin-like compounds, explaining its effectiveness in both spatial imaging and machine learning-based band selection. Thus, beyond its statistical significance, the selection of this wavelength is also supported by well-established vibrational spectroscopy theory.

To provide cross-method diagnostic evidence for the attribution of selected wavelengths to quercetin-related variance, we complemented SPA/Pearson/CARS selection with PLSR, NMF, and derivative-preprocessing diagnostics (Figure 3c–e) PLSR-derived VIP scores). Figure 3c identifies a cluster of wavelengths with VIP > 1, indicating above-average importance for concentration prediction in the standard series; notably the top VIP value is observed at 923.0 nm (VIP = 2.93). PLSR loadings for the primary predictive component are consistently positive across the examined band range, supporting a coherent contribution of these bands to the component associated with concentration variance. NMF of the leaf spectra recovered four component spectra with distinct shapes (Figure 3d) These components capture different spectral patterns across the near-infrared window; some components are relatively strong at shorter wavelengths while others increase toward longer wavelengths. The presence of an NMF component that resembles the average standard spectrum provides additional evidence that quercetin-like spectral variation is present in leaf measurements. Finally, derivative and scatter-correction preprocessing were tested for robustness (Figure 3e) The first derivative notably increased the linearity of the standard–cultivar correlation compared with raw spectra, whereas the second derivative introduced additional noise. These patterns indicate that the quercetin-associated bands are not artifacts of baseline or scattering, but remain consistently detectable under derivative preprocessing. Importantly, while PLSR, NMF, and derivative analyses provide convergent spectral evidence, they do not substitute for chemical verification. Definitive attribution of these spectral components to quercetin requires chromatographic assays (HPLC/UV or HPLC–MS) on the same material.

### 2.3. Transferability of the Standard Model to Plant Samples

**Figure 4:**
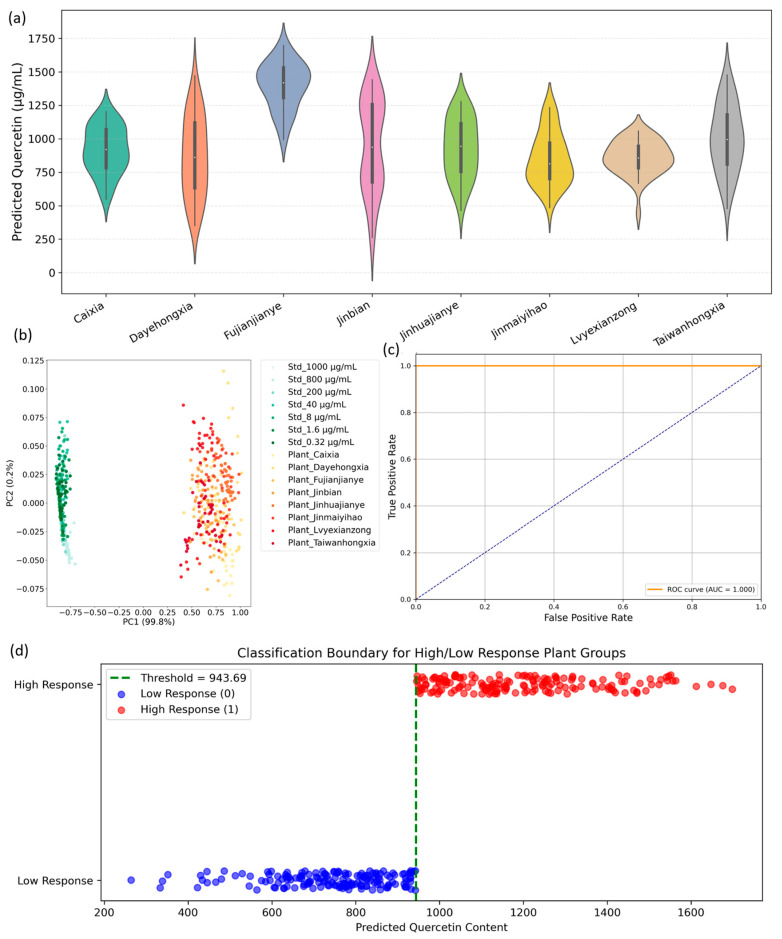
Model transfer and classification diagnostics linking standard quercetin spectra to leaf samples. (**a**) Violin plots of predicted quercetin concentration for eight *A. roxburghii* cultivars. (**b**) PCA score plot combining the standard dilution series and leaf spectra, showing a concentration-driven gradient and partial overlap between standards and plant samples. (**c**) ROC curve for the binary high/low response classifier. (**d**) Classification boundary for high/low response groups based on predicted quercetin content; the dashed vertical line indicates the chosen threshold (≈943.7 μg·mL^−1^) and points are colored by class.

Building upon the spectral characterization and band-specific feature extraction detailed in Section 3.2, the current study assesses the feasibility of applying a quercetin standard-trained regression model to the more complex hyperspectral profiles of *A. roxburghii* leaves. This step is critical for evaluating whether pure compound spectral fingerprints, acquired under controlled conditions, can be effectively transferred to heterogeneous plant matrices for semi-quantitative biochemical inference. As shown in Figure 4a, the pretrained model—trained solely on quercetin standard solutions—produces continuous predicted quercetin content values when applied to leaf spectra across eight cultivars. This demonstrates robust spectral transferability despite the inherent biological complexity and variability of plant tissues. Notably, cultivars such as Fujianjianye, Jinbian, and Taiwanhongxia exhibit significantly higher median predicted quercetin contents, with Fujianjianye showing a tightly clustered distribution centered near ~1450 μg/mL. This strong and consistent spectral alignment with concentrated standards suggests a comparatively elevated abundance of quercetin or chemically similar flavonoids in these cultivars. Conversely, cultivars including Jinmaiyihao, Caixia, and Dayehongxia display lower median values accompanied by broader or multimodal distributions, reflecting either reduced quercetin-related metabolite levels or increased biochemical heterogeneity within their leaf samples. The ability of the model to generate differentiated continuous outputs from complex leaf spectra underscores the utility of targeted SPA-selected wavelengths in capturing relevant quercetin-related spectral features, which persist despite the confounding effects of other biochemical constituents and leaf structural variability. Although absolute quantification was beyond the scope of this work, the observed relative response patterns align with expected biochemical differences and support the potential of hyperspectral imaging combined with pretrained models for rapid, non-destructive metabolic profiling.

The subsequent PCA (see Figure 4b) further substantiates these findings. Projecting combined standard and plant spectra onto the first two principal components—accounting for 99.8% and 0.2% of total variance, respectively—reveals clear spectral segregation. Standard solutions cluster tightly with minimal variance, indicative of spectral homogeneity, while plant samples occupy a distinct, more dispersed region along PC1. This separation highlights the additional biochemical complexity of natural leaf tissues compared to pure standards but confirms that SPA-selected bands effectively capture discriminative spectral variance relevant to quercetin-like compounds. Thus, PCA validates the transferability of key spectral features from standards to biological samples, providing a strong foundation for supervised predictive modeling. Exploring classification feasibility, predicted quercetin values were dichotomized into low- and high-response groups based on the median threshold (~943.69 μg/mL). The ROC curve (shown in Figure 4c) demonstrates perfect classification performance with an AUC of 1.000, indicating excellent sensitivity and specificity. Complementarily, the classification boundary visualization (Figure 4d) distinctly separates the two response classes with minimal overlap, reinforcing the robustness of the median-based threshold. These results collectively validate the practical applicability of predicted quercetin content for functional cultivar classification, which could serve as a foundation for phenotypic selection or quality control protocols.

From a broader perspective, integrating hyperspectral imaging with targeted feature extraction and pretrained standard models offers clear advantages for medicinal-plant metabolite analysis. It enables rapid, spatially resolved, and non-destructive assessment of bioactive compounds in situ and reduces reliance on laborious chemical assays. However, this approach also faces limitations, including potential spectral interference from co-occurring metabolites, leaf surface heterogeneity, and the absence of direct chemical calibration, which constrain absolute quantification accuracy. Future work incorporating reference quantifications from HPLC or other chemical assays would enable precise calibration and validation of predictive models, enhancing quantitation fidelity. Additionally, expanding spectral libraries and refining feature selection algorithms will further improve model robustness across diverse cultivars and environmental conditions.

Relative to many prior HSI studies that focus on bulk indices such as ‘total flavonoids’, our approach emphasizes band-level diagnostics anchored on a pure-compound dilution series. Previous reports (e.g., Tartary buckwheat, Ginkgo biloba, Chrysanthemum morifolium) demonstrate the feasibility of reflectance-based estimation for bulk flavonoid/phenolic pools, but these studies differ from ours in analyte focus, sample matrix, spectral windows and validation protocols [31,32]; consequently, they provide methodological context rather than direct numerical benchmarks. He et al. [25] extended this approach by integrating multivariate and deep-learning models with wavelength-selection techniques to simultaneously predict several micro-components in *C. morifolium*, achieving promising results for luteolin and quercetin. While these studies establish feasibility, they largely treat spectral models as black boxes. In contrast, the present study focuses on quercetin in *A. roxburghii* leaves and moves beyond prediction toward mechanistic interpretation. By combining a pure quercetin dilution series with PLSR-VIP analysis, Pearson/SPA feature selection, derivative-enhanced spectral correlations, and NMF decomposition, we systematically identified and corroborated hyperspectral bands linked to quercetin concentration, with a consistently influential band near 923 nm. This multi-pronged diagnostic strategy provides cross-validation of feature importance, supporting the chemical interpretability of the selected bands. Our methodological anchoring therefore differs from prior HSI studies: rather than maximizing predictive performance alone, we emphasize compound-specific spectral attribution and evidence of band-to-constituent transferability. At the same time, we recognize the limitations of this exploratory framework. The conclusions are based on spectral comparisons between pure standards and intact leaf spectra, and should be interpreted as relative evidence of spectral transferability. Absolute quantification of quercetin in plant tissues requires integration with chemical assays such as HPLC to calibrate models, verify concentration values, and evaluate prediction bias and accuracy. Such validation will be pursued in future work. Ultimately, this study establishes a promising technical pathway for digital, visualized, and non-destructive quality evaluation of *A. roxburghii* and potentially other traditional Chinese medicinal herbs. The demonstrated transferability of standard-trained hyperspectral models to complex biological samples provides a methodological foundation for advancing precision agriculture, breeding selection, and pharmaceutical quality assurance through hyperspectral technology.

## 3. Materials and Methods

### 3.1. Plant Materials and Standard Solutions

**Figure 5:**
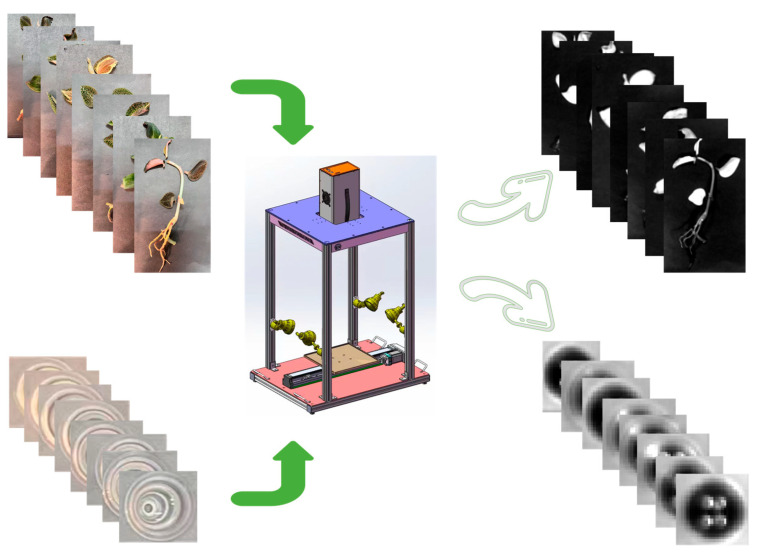
Overview of experimental materials and spectral acquisition. Top left: photographs of representative *A. roxburghii* cultivars. Top right: leaf reflectance spectra. Bottom left: quercetin standard solutions. Bottom right: spectral profiles of quercetin solutions. Center: hyperspectral imaging system used for data collection.

Fresh leaves from eight cultivars (Figure 5) of *A. roxburghii*—Caixia, Dayehongxia, Fujianjianye, Jinbian, Jinhuajianye, Jinmaiyihao, Lvyexianzong, and Taiwanhongxia—were obtained from the Department of Traditional Chinese Herbal Medicine, Zhejiang A&F University. For each cultivar, 10 healthy and mature leaves were collected from the middle canopy. Samples were gently rinsed with distilled water to remove surface particulates and air-dried using lint-free paper. Hyperspectral imaging was performed immediately after sample preparation to minimize moisture loss and biochemical degradation.

Quercetin (≥98% purity, Yuanye, Shanghai, China) was used as the chemical reference standard. A stock solution (1000 μg/mL) was prepared by dissolving quercetin in 70% ethanol–water. To construct the calibration/working series for the quercetin standard, we prepared solutions at 0, 0.32, 1.6, 8, 40, 200, 800 and 1000 μg·mL^−1^. The series includes a zero (blank) for baseline correction and low-concentration points to assess the method’s limit of detection/quantification and signal-to-noise at the low end. Intermediate points follow an approximately geometric spacing (≈5× steps) to provide even coverage across a broad dynamic range (≈0.32–800 μg·m^−1^, ≈3.4 orders of magnitude), which facilitates robust calibration across both low and high concentration regimes while keeping the total number of standards practical. The highest concentrations (800 and 1000 μg·m^−1^) were included to probe potential nonlinearity or detector saturation and to evaluate matrix- or concentration-dependent spectral changes. Standards were prepared by serial dilution from a single concentrated stock to minimize volumetric error and ensure consistency across the series.

### 3.2. Hyperspectral Imaging System and Acquisition

Hyperspectral imaging was performed using a line-scan push-broom system (Dualix Spectral Imaging, Chengdu, China) operating in the NIR range. The system consisted of a high-resolution InGaAs camera (640 spatial pixels, 512 spectral bands), an imaging spectrograph covering 900–1700 nm (spectral resolution: 5 nm), and four 50 W halogen lamps symmetrically arranged to ensure uniform diffuse illumination. Image acquisition and hardware control were conducted using SpectraVIEW V1.0 software. Prior to scanning, the system was warmed up for 30 min. Calibration images included white reference scans (using a >99% reflectance standard panel) and dark current scans (acquired with the lens cap on). Samples were carefully placed on a motorized conveyor belt with a matte black background to reduce stray reflections. The belt speed was set at 0.6 cm/s, synchronized with the frame rate to maintain consistent spatial resolution. Exposure time was optimized to avoid pixel saturation across the spectral range. Both leaf samples and liquid standards were scanned under identical conditions. Each hyperspectral image was acquired as a three-dimensional data cube, with raw data stored as digital number values for subsequent calibration.

### 3.3. Image Preprocessing and Region of Interest Extraction

All raw hyperspectral images were radiometrically corrected to reflectance values using the standard formula: *R* = (*I_raw_* − *I_dark_*)/(*I_white_* − *I_dark_*). Where *I_raw_*, *I_dark_* and *I_white_* denote the raw, dark, and white reference images, respectively. This correction eliminated sensor dark current noise and normalized lighting variations.

ROIs were selected to extract representative spectra. For plant leaves, ROIs were manually or semi-automatically delineated within intact, non-vein regions using ENVI 5.3 software. For standard solutions, ROIs were selected from the central, homogeneous portion of each liquid surface to minimize boundary artifacts. The mean reflectance spectrum from each ROI was computed, yielding 40 spectra per cultivar or concentration group. To quantify measurement repeatability and within-group variability, each ROI extraction produced *n* = 40 replicate spectra per cultivar or concentration group. For spectral reporting, we calculated the sample mean (µ) and standard deviation (SD) at each wavelength across the replicates. The coefficient of variation (CV, %) was computed as CV = (SD/µ) × 100 to express relative variability. For model stability assessment, we report the mean and SD of model performance metrics (R^2^, RMSE and RPD) across five-fold cross-validation. These reproducibility metrics were used both to annotate spectral plots (mean ± SD) and to populate the supplementary reproducibility table, which lists mean, SD and CV for each SPA-selected wavelength by standard concentration and by cultivar.

### 3.4. Data Analysis Methods

Hyperspectral data analysis was conducted through a combination of spectral preprocessing, dimensionality reduction, feature selection, regression modeling, and spectral transfer validation. All analyses were performed using Python 3.9, with key packages including scikit-learn, scipy, TensorFlow, and Matplotlib.

The reflectance spectra of standard quercetin solutions were first visualized by plotting mean ± standard deviation curves across concentrations. Principal Component Analysis (PCA) was applied to the full preprocessed dataset to assess variance structure and identify potential clustering.

Three feature selection methods were employed to identify wavelength variables with the highest relevance to quercetin concentration. (i) Pearson correlation analysis retained wavelengths with absolute correlation coefficients |r| > 0.6. (ii) Successive Projections Algorithm (SPA) was used to extract 10 non-collinear, informative bands with minimal redundancy. (iii) Competitive Adaptive Reweighted Sampling (CARS) was applied with 50 Monte Carlo runs and a 10% variable retention rate per iteration, using regression coefficients from Partial Least Squares models to rank importance.

Based on SPA-selected bands, a one-dimensional convolutional neural network (1D-CNN) was constructed for regression. The network consisted of an input layer, a 1D convolutional layer (64 filters, kernel size = 5, ReLU activation), a max-pooling layer (pool size = 2), a flattening layer, a dense layer (32 units, ReLU), and a linear output node. The model was implemented in Keras with the Adam optimizer and trained using mean squared error loss. Five-fold cross-validation was used to evaluate model performance, with R^2^ and residual predictive deviation (RPD) as primary metrics.

For visualization, reflectance intensity at representative SPA-selected wavelengths (e.g., 923 nm) was mapped to false-color images to explore spatial spectral distributions. Spectral similarity between plant cultivars and standard concentrations was evaluated by computing the Pearson correlation coefficient between their mean spectra, restricted to SPA bands. The resulting similarity matrix was visualized as a heatmap.

In order to strengthen attribution of selected wavelengths to quercetin-related spectral variance, feature selection on the standard series was complemented by several diagnostic analyses. Specifically, (i) Partial Least Squares Regression (PLSR) loadings and Variable Importance in Projection (VIP) scores were inspected to confirm whether bands selected by SPA/Pearson/CARS also exhibit high PLSR importance; (ii) simple spectral unmixing (non-negative matrix factorization, NMF) was applied to leaf spectra to detect component spectra that resemble the pure quercetin standard profile; and (iii) derivative preprocessing (1st/2nd derivatives) and scatter-correction (SNV/MSC) were tested to check the robustness of selected bands against baseline and scattering artifacts. These complementary diagnostics were not intended to replace chemical assays, but to provide cross-method spectral evidence that the selected wavelengths consistently capture concentration-related variance in standards and manifest as coherent components in leaf spectra.

The pretrained 1D-CNN model was subsequently applied to spectra from *A. roxburghii* leaf ROIs to generate predicted quercetin values. These predictions were aggregated per cultivar for comparative analysis. PCA was performed on the combined spectra of standard and plant samples using only the SPA bands to evaluate spectral domain overlap.

For binary classification, the median predicted concentration across all plant samples was used as a threshold to define high- and low-response groups. A Receiver Operating Characteristic (ROC) curve was generated, and the Area Under the Curve (AUC) was calculated. A classification boundary plot was also constructed to visualize group separation.

## 4. Conclusions

This study demonstrates that near-infrared hyperspectral imaging combined with band-specific feature analysis and a standard-trained 1D-CNN can detect quercetin-related spectral variance in A. roxburghii leaves and enable semi-quantitative, spatially resolved mapping under our experimental conditions. Key wavelengths (notably ~923 nm) consistently emerged across multiple diagnostic methods and supported the spectral transfer from pure standards to intact leaf spectra. Limitations of the present model system include its dependence on the specific instrument and preprocessing pipeline, potential spectral interference from co-occurring metabolites and tissue heterogeneity, the limited number of cultivars sampled, and the absence of chromatographic calibration on the same leaf material; therefore, absolute quantification is not claimed. Future work will integrate chromatographic validation, expand cultivar and environmental sampling, develop calibration-transfer and multi-sensor strategies, and pursue field-level validation to improve robustness and enable practical, quantitative hyperspectral screening for medicinal-plant quality control.

## Figures and Tables

**Table 1:** SPA-selected wavelengths and their Pearson correlation coefficients with quercetin concentration (absolute r and |r|).

Wavelength (nm)	Pearson r	|r|
967.3	0.543	0.543
1638.7	−0.185	0.185
1192.9	0.16	0.16
1351.9	0.29	0.29
1403.4	−0.134	0.134
1235.8	0.253	0.253
923	0.696	0.696
1135.1	0.594	0.594
1536.7	−0.165	0.165
1176.3	0.365	0.365

## Data Availability

The full dataset can be accessed upon reasonable request.
